# Restoring Symmetry and Enhancing Exchange via Chiral Molecular-Magnetic Hexagons

**DOI:** 10.1021/acs.jpca.5c05598

**Published:** 2026-02-19

**Authors:** Mark R. Pederson, Zahra Hooshmand, Difan Zhang, M. F. Islam, Kushantha P. K. Withanage

**Affiliations:** † Department of Physics, 12337 University of Texas at El Paso, El Paso, Texas 79968, United States; ‡ 6146 Pacific Northwest National Laboratories, Richland, Washington 99354, United States

## Abstract

Modified behaviors of molecules, designed for device or energy applications, can occur due to lattice-molecule incompatibilities in point-group symmetries (PGS), long-range interactions between neighboring molecules, or lattice-molecule charge transfer. Such sensitivities are a prerequisite for the use of quantum molecules as devices, but removing certain broken symmetries is desirable from the standpoint of simplifying their behaviors. Here, we demonstrate symmetry restoration to molecular-magnetic lattices and show that it leads to strengthened exchange coupling due to dipolar electrostatic interactions between neighboring molecules and due to manifestations of an earlier identification of a spin-sensitive slightly mobile electron by et al. [ HooshmandZ.,  Phys. Rev. B 2021, 104, 134411]. Starting with a broken symmetry molecular magnet of current interest, we demonstrate a more robust structure composed of six molecular magnets on an *h-*BN-like surface. The broken point-group symmetry is healed by constructing interpenetrating equilateral triangular structures that are appropriately ratcheted to preserve 3-fold rotational symmetry and inversion symmetry.

## Graphical abstract

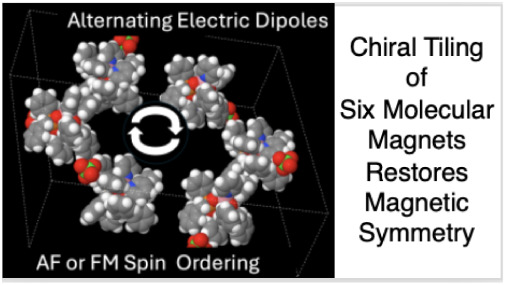

## 1 Introduction

In ref 1, Johnson and coworkers showed how cyclical tori-like structures could be computationally synthesized through the use of point-group symmetry. Of direct relevance to the work reported here, this methodology showed how to take structures that were intrinsically asymmetric and build new structures with quasi-cylindrical symmetry. Here we use this point of view to create chiral structures that can be assembled on hexagonal lattices.

The coexistence and interdependencies associated with chirality and spin in chemical systems have been of long-term interest to chemistry, biology, and physics, and are currently receiving accelerated attention. For example, Naaman and Waldeck and other researchers have published work highlighting the so-called chiral-induced spin-selectivity effect.^2–6^ Islam et al.^7,8^ have demonstrated the emergence of chiral spin eigenstates in molecular magnets composed of 3 metal centers that are protected by ligands, and additional theoretical and experimental studies have shown that perfect 3-center systems^9^ can be realized. While molecular magnets are reasonably well protected by their ligands, a goal for the future is to determine how trimers or hexamers composed of molecular magnets, rather than single metal centers, can be assembled and safely protected from their environments. Prospects for building molecular structures that resemble toroids have fascinated scientists for at least 30 years.

One of the earliest density-functional calculations that employed five- and six-symmetry operations (PGS) to create toroidal systems with a small number of inequivalent carbon atoms was introduced by Johnson et al.^1^ Here, we use the same techniques to create ionically bound slightly disconnected toroids from a previously synthesized bulk array of molecular magnets.^11^ In this work, it was shown that by choosing a point-group symmetry and a toroidal radius, one could determine how to glue small carbon fragments together by allowing the rotated fragments to form covalent bonds with one another or the original fragment. That work was also partially inspired by the close relationships between carbon nanotubes, and the experimental existence of such nanotubes provided a degree of confidence that toroids would be synthesized in the future, despite the fact that the two structures are not identical. Examination of references to that paper shows that the degree of computational and theoretical interest significantly outweighed experimental realizations for at least a decade despite the uniform acceptance of π-bonding by carbon. An early experimental example in molecular magnetism, by Tasiopoulos et al.,^12^ reported the synthesis of a Mn_84_ structure that occurred as part of the attempt to fuse Mn_12_–Acetate molecules. Similarly, Mahmood et al.^13^ have computationally studied the possibility of making porphyrin rings (synthesized by Anderson et al.)^14^ and discussed the possibility of forming magnetic currents around the rings.^13^ Indeed, while the early computational aspirations^1^ were primarily confined to carbon-based structures, literature searches show that the ideas are now common in both organic and inorganic systems, and there is every reason for computational scientists to emulate the curiosity-driven computational approach of Johnson et al.^1^ to slightly, but not literally, mimic known experimental structures.

A case where a crystal of a *Co*_2_-based molecular magnet that as a single unit does not have quasi-cylindrical symmetry (Maass et al.)^11^ is discussed here. We show that the use of point-group symmetry leads to a chiral hexagon that might be better described as a disconnected toroid. However, one can imagine connecting the monomers with bridging covalent bonds or an ionic scaffold to stabilize it as a toroid.

The goal of computationally accelerating the design of isolated molecular or cluster-assembled magnets, informed by infinite periodic arrays of such systems, is long-standing.^15–21^ Full success requires ensuring compatibility, in terms of point-group symmetry (PGS) and relative electronegativities, between future adsorbed molecular devices and surfaces. In this regard, compatibility with hexagonal surfaces^22^ is especially important due to their ubiquity. While notable and ideal counterexamples exist (e.g., Mn_12_–Acetate which shares neutral molecules of crystallization but no counterions with its neighbors), it is most often the case that for a bulk crystal containing an N-fold axis, counterions or molecules of solvation that appear in the solid are shared equitably by N magnetic molecules with each molecule having N nearest PGS-preserving counterions. As such, preserving both the symmetry and the overall neutral charge states of the magnetic-molecule-counterion system cannot be achieved by only including one of the N equivalent counterions. Further addressing (e.g., reading or switching) a single molecular magnet in any system with the extremely short nanomagnet–nanomagnet separations found in crystals is a huge challenge of its own, which further highlights the need to be able to design individual molecular magnets or conglomerates prior to the well-spaced deposition of the resulting qubits. It is therefore the role of computation to determine whether and how a technologically useful isolated mimic of the bulk idealization of the moiety can be built. Because quasi-cylindrical symmetry is a figure of merit for such systems, this necessarily means that one cannot naively adopt the literal structure obtained from an X-ray analysis. In Figure 1, we consider an intrinsically broken-symmetry (PGS) charge-compensated 137-atom ″Co_2_″ molecular magnet as an example and show how it can be used to build a larger fully symmetric, albeit chiral, (Co_2_)_6_ qubit.

**Figure 1:**
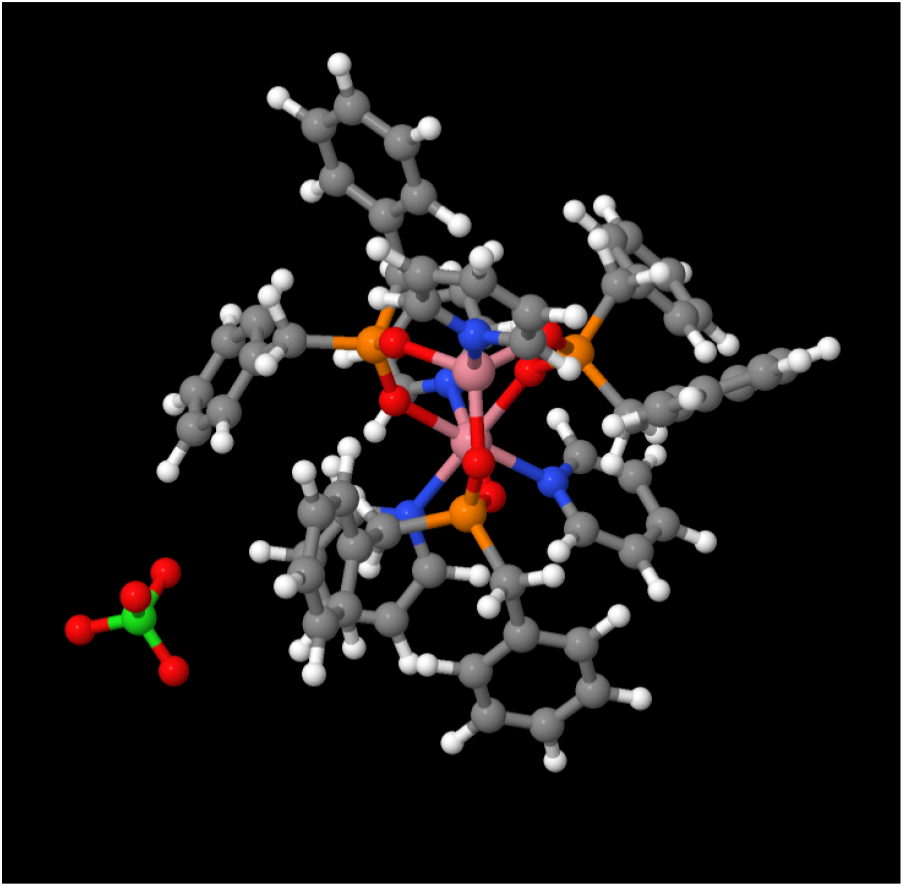
L-[*Co*_2_]­(*ClO*_4_) system studied here. As synthesized, the terminating planar Ligand (L = *NC*_5_ *H*_5_) and noncoaxial perchlorate break the otherwise perfect 3-fold symmetry of the system.

Motivated by experimental developments on a molecular magnetic crystal by Maass et al.,^11^ we perform calculations on the L-[*Co* ^+2^(μ_2_ *PO*_2_(*H*_2_ *CC*_6_ *H*_5_)_2_]^−1^)_3_ *Co* ^+2^(*C*_5_ *H*_5_ *N*)_3_]­(*ClO*_4_)^−1^, L = *C*_5_ *H*_5_ *N* and L = *NH*_3_ systems. An isolated relaxed geometry for this system is shown in Figure 1. Hereafter, we refer to the entire complex as Co_2_, and the explicitly 3-fold symmetric part of the complex, in red, as [Co_2_]. Curiously, each of the three bridging ligands between the two cobalt double cations has a negative charge. Together with the single perchlorate anion, these compensate for the overall charge of the system, but at least one electron is shared between the two ligands, which complicates the electronic structure at the Fermi level and leads to the sensitivity in FM-AM ordering.^23^

### 1.1 Aspirational Ammonia-Terminated Mimic

We first present the simplest, most ideal target system that has been inspired by the synthesis of the molecule exhibited in Figure 1 and then discuss conglomerated systems that would not require ligand substitution or anion repositioning. Examination of the system shows that much of the molecule is compatible with a 3-fold symmetry axis, but the nearest-neighbor counterions and a terminating pyridine ligand break this point-group symmetry. An aspiration based on the actual synthesized periodic bulk crystal would be to replace the symmetry-breaking pyridine with *NH*_3_ and move the perchlorate anion, or other anions with 3-fold symmetry, so that it is coaxial with the 3-fold axis of [*Co*_2_]. Our predicted results, labeled as case A-1, are summarized in Table 1. Restoring 3-fold axial symmetry through the described ligand substitution and counterion movement yields an FM molecule with a huge MAE due to the half-filled *e*_*g*_ HOMO level. As discussed in ref 10, when the spin coupling is switched to AF, the energetic ordering of the 2-fold HOMO and LUMO is switched which removes a degeneracy at the Fermi level and then turns off the large magnetic anisotropy. However, in both cases, the goal of creating a system that is uniaxial is achieved. In contrast for the as-synthesized pyridine-terminated structure, even without the counterion, the 2-fold symmetry is broken, which lifts the possible degeneracy between *e*_*g*_ HOMO levels and leads to significantly lower easy-axis anisotropy with appreciable transverse anisotropy terms (case E-1 in Table 1).

**Table 1:** Cases A-0 and A-1 Show the Volatility in the HOMOs and LUMOs Due to Changes in Spin Ordering^a^

Case	Magnetic Molecule	Order	MAE	E/D	Gap
A-0	(*H*_3_ *N*)[*Co*_2_]^+^	AF	15	0	0.55
	(Isolated Molecule)	FM	416	0	0.0
A-1	$\left(H_{3}N\right){[Co_{2}]}^{+q}ClO_{4}^{-q}$	FM	10	0	0.0
A-2	$\{\left(H_{3}N\right){[Co_{2}]}^{+}ClO_{4}^{-}{\}}_{6}$	AF	55	0.0	0.54
	(Isolated Hexagon)	FM	55	0.0	0.54
A-3	${[\{(H_{3}N){[Co_{2}]}^{+}ClO_{4}^{-}{\}}_{6}]}^{-3}$	FM	273	0.0	0
E-1	(*C*_5_ *H*_5_ *N*)[*Co*_2_]^+^	AF	23	0.08	0.0
		FM	–78	0.06	0.0
E-2	$\left(C_{5}H_{5}N\right){[Co_{2}]}^{+}ClO_{4}^{-}$	AF	–13	0.63	0
		FM	–13	0.03	0.25
E-3	$[\left(C_{5}H_{5}N\right){[Co_{2}]}^{+}{\left(ClO_{4}\right)}^{-}{]}_{6}$	AF	–59.3	0.0	0.64
		FM	–59.6	0.0	0.65

a For the monomer, this is further complicated by incomplete charge transfer, within DFT, to $ClO_{4}^{-q}$ . Table includes magnetic anisotropy (MAE) in Kelvin, the ratio of transverse to longitudinal anisotropy , and the HOMO-LUMO gap (eV) as a function of spin ordering and the number of monomers . A negative sign of the barrier indicates an easy plane. A positive sign indicates an easy axis . Results on monomers are from ref^10^. The strong reduction in MAE in the hexagon (A-1) relative to the monomer (A-0) identified by Hooshmand et al.^10^ is due to a crossing of the two-fold and one-fold electronic states that quenches the permanent orbital moment . To test this , we also perform rigid-band calculations on the trianion. For this case , the partially occupied states lead to large anisotropy.

## 2 Symmetrized Chiral Molecular Magnetic Qubits

The use of well-separated, point-group-symmetry-preserving molecular assemblies is expected to provide the possibility of strong magnetism or quantum devices, achieved from simple transition-metal dimers^24,25^ but requires strategies for protecting the magnetically interesting ions from their environment. Two-dimensional hexagonal lattices of BN and graphene, while well studied, either as separate lattices or by stacking one-dimensional films atop one another, offer templates for approaches to building more complex lattices in which the boron and nitrogen sites may be substituted by complex molecules. Early and recent works on such lattices range from refs 26,27 .

Separate sequential design of surface templates and cluster assemblies is common, but we suggest here that a better approach could involve choosing molecular magnetic moieties in a manner that allows groups of the adsorbates to actively participate in defining the symmetry of the resulting surface template. We start by considering an h-BN lattice (see Figure 2) but with one and only one inequivalent molecular moiety, carrying internal geometrical, spin, and electronic structure, that is placed in variable orientations on either the B or N sites. It is further assumed that the ordering of the spin degrees of freedom, i.e., antiferromagnetic (AF) vs ferromagnetic (FM), is either correlated with or possibly even determines the dipole moment of the molecule. If the same molecule is placed on the B site but with a symmetry rule that, at the minimum, includes a rotation by $\frac{2\pi }{6}$ relative to the N site, it is possible to derive a graphene-like lattice^22^ composed of otherwise identical moieties that are arranged in well-defined rotations relative to one another on a standard graphene lattice. However, within the stated assumption that the molecular internal degrees of freedom define the system’s electric dipole, this construction leads to a graphene-like structure with aligned electric dipoles that would then repel one another. Instead, if the *h*-BN lattice is derived by turning every other molecule upside down, then a lattice of these molecules could be held together by relatively strong nearest-neighbor dipole–dipole attractions, as is the case for a BN lattice. Possibly with additional modifications of the two sublattices, anion–cation attractions associated with the standard *h*-BN lattice could be achieved through doping of the [*Co*]_2_. Further, if within the parent molecule, hereafter referred to as the molecular magnetic building block (MMBB), interdependencies between spin ordering and dipole moment are strong enough, the apparent exchange coupling within any molecule on the lattice could be significantly strengthened because energetic changes of spin ordering would then be linked to long-range Coulombic interactions rather than standard superexchange interactions.

**Figure 2:**
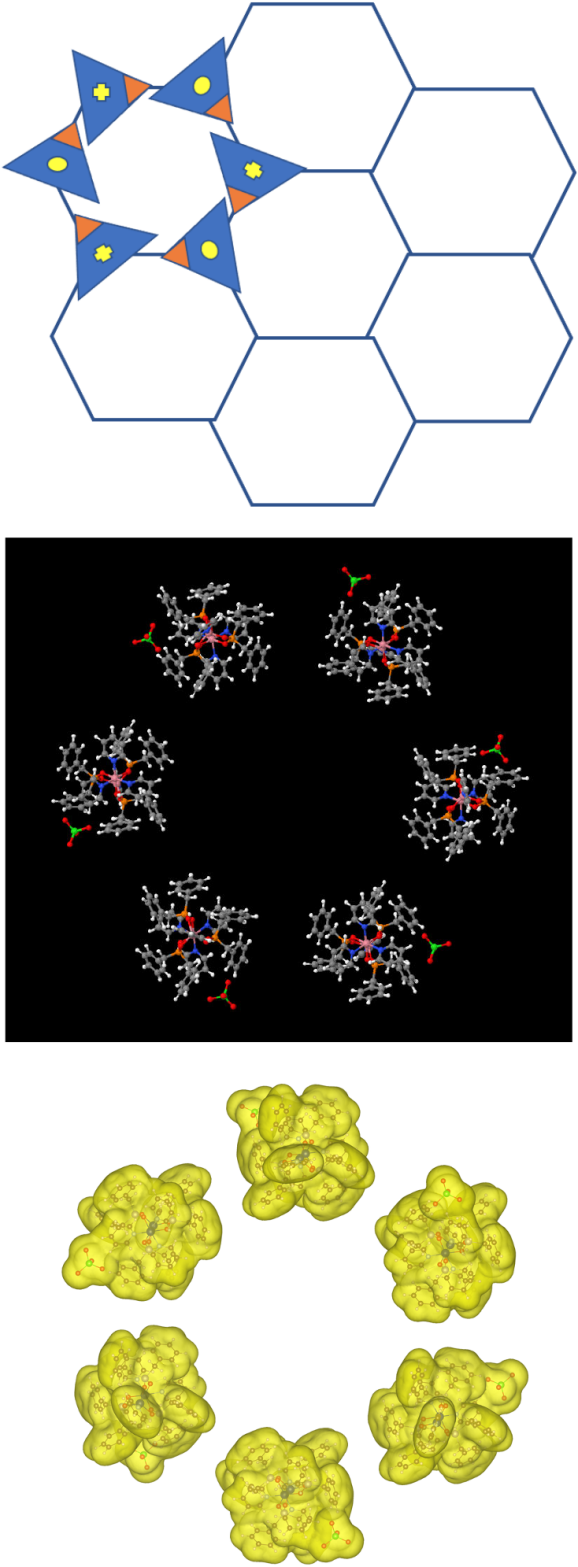
Broken-symmetry (PGS) MMBB, induced by the ligand and counterion, when self-assembled on a hexagonal lattice restores the desired quasi-cylindrical symmetry due to the preferential energetic ordering of the MMBB dipoles. Nearest neighbor molecules are rotated by $\frac{2\pi }{6}$ and then reflected through the plane leading to antiparallel neighboring electric dipoles (yellow dot/cross in the top panel). The hexagonal qubit has an MAE of 75 K (easy plane with a nearly vanishing HOMO–LUMO gap) and shows sensitivity to FM vs AF on-molecule spin coupling. The broken-symmetry orange and blue triangles represent the local point-group symmetry-breaking role of the displaced counterions and pyridine ligands in the actual molecule.

To quantify this further using a point-charge model, (+1) at the center and (−1) on the perchlorate, we find that the dipole moment per *Co*_2_ in the hexagon is on the order of 17 atomic units for the pyridine-terminated structure. This is slightly smaller than the dipole moment calculated from PBE-GGA (20.2 atomic units). This leads to a net stabilization of approximately 0.265 eV. At the same geometry, the FM structure is more stable by 0.163 eV. If this energy were due to standard spin–spin coupling (Δ_*E*_ = 6 · 2 · (3/2)^2^ J), it would suggest a FM J of 6 meV.

There have been a significant number of calculations on triangular molecular magnets composed of spin 1/2 centers. Islam et al.^7,8^ and Trif et al.^28^ demonstrated apparent changes in spin coupling due to spin-electric effects which are now a well-accepted strategy for quantum devices. Such systems are interesting since the absence of inversion symmetry allows an external electric field to couple directly with the spin chirality that characterizes their ground state^29–33^ or systems composed of larger numbers of spin centers that reduce to three-spin systems at low temperatures such as V_15_.^17^ For AF triangular arrays, there are often four degenerate states that can be decomposed into two sets of accidentally degenerate chiral and antichiral states. It has been shown by Nossa et al.^30^ that weak spin–orbit effects due to coupling between the singly occupied systems and the doubly occupied systems can split these Kramers’ doublets, albeit very weakly. It is possible that the stronger structural chirality associated with the toroids discussed here could more strongly isolate the lower Kramers’ doublet, which could help achieve the goal of using three-center triangular molecules as qubits.

Alternatively, the triangular sublattices may also be used as potential spin-electric system effects, with one very important distinction. Again, in this case, it would be structural effects that lead to chirality, and these could provide much stronger splittings in the ground-state structures. The general picture described here and motivated by these observations provides a basis for searching for a class of low-density transition-metal or rare-earth hexagonal systems with enhanced exchange couplings and huge anisotropies.

### 2.1 Hexagonal Assemblies

In our earlier calculations on the isolated system, we found that the expected MMBB-to-perchlorate electron transfer is partial rather than complete, as is often found in donor–acceptor pairs within density-functional calculations. However, in calculations on the 864-atom complex shown in Figure 2, full charge transfer is achieved due to the Madelung stabilization associated with the alternating dipole moments. The sensitivity associated with the slightly mobile electron suggests that full SCF calculations are required to clearly determine how the on-molecule dipole moment reacts with the environment and the Co–Co spin coupling. When we self-consistently determine the exchange coupling by calculations on the 864-atom complex, we find that the exchange coupling parameter reverses its sign due to differing AF/FM dipole moments. In contrast to results from standard exchange-coupling-constant calculations which are exaggerated by slightly delocalized d-electrons within standard DFT (see, for example, Ruiz et al.^34,31^ ), the predicted exchange coupling energies here are unlikely to be overestimated since they are driven by Coulomb interactions rather than the more standard mechanisms described by Goodenough.^35^

Beginning in 1934 and culminating with papers by Goodenough and Kanamori,^35–37^ researchers have noted that the sign of coupling between second-nearest-neighbor metal ions is mediated by 3*d*/2*p* mixing between the ion pairs and their shared first-neighbor ligands (often oxygen-like). Essentially due to the Pauli principle, the resulting superexchange interaction is antiferromagnetic in cases where the resulting frontier ionic d-electrons are orthogonal by symmetry and antiferromagnetic when the frontier ionic d-electrons are not orthogonal by symmetry. We note that the interactions controlling AF vs FM coupling of second-neighbor ions discussed here are determined by kinetic-energy repulsions between neighboring ions for the case of standard coupling and are controlled by changes in local electric dipoles in the second case. These effects are well within the accuracy of mean-field couplings. It has been noted in many publications that the GK exchange couplings are often overestimated, in the case of DFT, when self-interaction corrections or hybrid methods are not included. This is due to the fact that the ionic d-electrons are too diffuse.^17^

Additional discussions on issues related to overly diffuse d-states, arising from density functional approximations, can be found in papers by Ruiz et al.^34,31,38^

The symmetry restoration idea, discussed here in terms of six equivalent slightly disconnected molecules is formally analogous to how symmetry plays a role in single-molecule magnets (Mn_12_–Acetate^15,39^ and Co_4_(hmp)_4_(CH_3_OH)_4_Cl_4_ ^40,41^ ). For each of these cases, the molecule can be generated from symmetry (both have *S*_4_) operations on a single asymmetric cluster containing one-fourth of the atoms. This is explicitly discussed in ref 41 for Co_4_ and in refs 15,42 for the Mn_12_–Acetate case. For the Co_4_(hmp)_4_(CH_3_OH)_4_Cl_4_ molecular magnet, where hmp is deprotonated hydroxymethylpyridine, the use of the PBE-GGA method has been partially validated by comparing PBE-GGA results^41^ to experiment.^40^ The Co atoms for the system studied here and the Co_4_ molecular magnet each sit in a local, albeit symmetry-broken, *D*_3*d*_ geometry, so conclusions drawn from comparing experiment and theory for that system are likely to carry over to this work. The PBE-GGA functional used there confirmed the experimental explanation that an overall easy axis, observed experimentally, was due to the orthogonal hard-axis alignment model but also showed that relaxation of the pyridine ligands in the isolated structure decreased the anisotropy relative to the experiment. From the standpoint of dependence on functionals, Fitzhugh et al.^43^ have tracked changes in exchange-coupling parameters as a function of 14 different functionals, transition-metal oxidation states, and local moments which provides an excellent survey of functional dependencies. Fitzhugh et al.^43^ considered global hybrid functionals with a fixed admixing parameter, six local hybrid functionals with spatially dependent admixtures, the SCAN^44^ and r^2^SCAN^45^ meta-generalized gradient approximations (GGAs), and two widely used GGAs. The latter functionals trended slightly toward an overcorrection in the error in magnetic coupling parameters relative to that of the PBE-GGA. The performance of local hybrid density functionals did not show improvement. Fitzhugh et al.^43^ concluded that more efforts are needed for the extension from global to local hybrid density functionals for exchange-coupling parameters. While this study suggested that SCAN and r^2^SCAN meta-GGAs may be the most reliable based on eight complexes studied in that work, the work also showed that PBE-GGA provided exchange-coupling parameters that were approximately twice the size of experiment and SCAN or r^2^SCAN. Generally speaking, magnetic anisotropies are expected to be accurate if exchange interactions are large enough to prevent reorientation of individual local moments. Here we posit that the relative decrease in anisotropy observed in the systems studied theoretically^41^ and experimentally^40^ suggests that the ions are more susceptible to departures from 3-fold symmetry in PBE-GGA treatment than in experiment. However, in ref 41, it was postulated that it was both internal pressures due to crystallization and the descissoring of the hmp ligands in the isolated structure. An alternative suggestion is that additional localization of the 3d-electrons, which would result from self-interaction corrected orbitals, could play a role in diminishing the ability of the electrons to observe the local 3-fold symmetry splitting.

The stronger dipolar interactions observed here further confirm a degree of electrostatic volatility, which provides interesting possibilities for electronic sensing and electrical control of the Co_2_ magnetic state.

The demonstrated ability to synthesize such molecules in hexagonal lattices (S_6_) along with the ability to alter the magnetic properties through manipulation of individual electrons, spin coupling, or ligand choice suggests that further investigation of this quantum magnetic system could be of interest to sensing or computing applications.

### 2.2 Symmetry Considerations for Isolated and Periodic Tiling

For any molecular moiety, it is possible to make a highly symmetric hexagonal lattice through groups generated by the following proper (+) or improper (−) rotations:

1 $$R=\left[\begin{array}{ccc} \cos\left(2\pi /6\right) & \sin\left(2\pi /6\right) & 0.0\\ -\sin\left(2\pi /6\right) & \cos\left(2\pi /6\right) & 0.0\\ 0.0 & 0.0 & \pm 1 \end{array}\right]$$

and the standard nonorthogonal lattice translations associated with a hexagonal lattice. For the improper case of interest here, prior to the translation by *T* = *n*_1_ *t*_1_ + *n*_2_ *t*_2_, the molecule is either properly (*n*_1_ + *n*_2_ even) or improperly (*n*_1_ + *n*_2_ odd) rotated by $R^{n_{1}+n_{2}}$ which leads to a hexagonal lattice composed of antiparallel nearest neighbor dipoles as shown in Figure 2. Within these fully periodic single-molecule structures, concerted spin-flips may also be included, which still preserve the high symmetry of the lattice as a whole. For example, a chosen $Co_{2}^{B}Co_{2}^{N}$ spin configuration may either alternate, e.g. (↑,↓) → (↓,↑), between nearest neighbors or be exactly preserved without changing the magnitude of the electric dipoles for each site. Both of these tiling schemes would lead to interpenetrating triangles which independently rotate into one another under rotations of $\frac{2\pi }{3}$ . This lattice has different characteristics than the S = 3/2 Kitaev lattices. Rather than creating reflections through a plane that bisects a hexagonal side, we instead rotate the molecule through $\frac{2\pi }{6}$ degrees and simultaneously reflect the molecule through the plane.

The energetic differences between the helical hexagonal toroid proposed here and the Kitaev structure depend on whether the building block has a dipole moment. For one idealization in which the dipole moments are perpendicular to the plane, it is energetically advantageous to have the nearest-neighbor dipole moments arranged antiparallel to one another. Similarly, if the molecular building block were to have an in-plane dipole moment, the helical structure proposed here would also lower the Coulombic energy relative to the Kitaev structure. On the other hand, for building blocks that have no dipole moment and an odd number of delocalized electrons, there could be the possibility of choosing reflection symmetry through a covalent dimerization of the pairs of mobile electrons.

### 2.3 Methodology

The all-electron calculations presented are based on the PBE-GGA correlation functional^46^ using methods that are well tested for molecular magnets and described in refs 15,47,48 . The molecular geometry for our MMBB was fully optimized as an isolated unit to fully account for the pyridine- and perchlorate-induced breaking of on-molecule 3-fold rotational symmetry. The hexagonal geometry was also optimized to realistically determine the nearest-neighbor separation in our model system. High-quality basis sets have been used^49^ and have been carefully tested by Park et al. in applications to molecular magnets.^50^ Discussion of the calculation of exchange coupling and magnetic anisotropies may be found in ref 48. The exchange coupling between a pair of cobalt ions in any one of the MMBB is antiferromagnetic.

Each molecule has two degenerate AF-coupled configurations (↑↓,↓↑) and two degenerate FM-coupled configurations (↑↑,↓↓). Two different intramolecular dipole moments could arise from the AF and FM configurations. Ref 10 found that the transition from the AF ground state to the FM excited state was indeed associated with a change in electronic configuration on Co^*A*^. This may lead to a variation in FM versus AF dipole moments, although not a large one.

For a given hexagon, there are then 4^6^ low-energy spin arrangements that can be constructed. For the system discussed here, there are two different Co sites that each carry a spin $S=\frac{3}{2}$ . In a calculation where we ask for the energy as a function of universal spin orientation, all spin degrees evolve collectively, leading to a Hamiltonian that describes resonant tunneling of magnetization and single-spin behavior.^15^ As expected on general grounds and as verified in well-studied molecular magnetic systems, the idealized behavior, valid at low temperatures, is modified when noninfinite exchange coupling is included. Irrespective of the size of the exchange coupling, but within the *ansatz* that the spinors associated with a many-electron wave function or set of Kohn–Sham orbitals evolve under a specific rotation, an accurate magnetic anisotropy may be derived by determining how the spatial orbitals respond perturbatively or exactly to the spinor rotations. An effective, but not perfect, single-spin Hamiltonian then describes the system’s behavior at temperatures that are low compared to the anisotropy energy.

Before continuing, we note that the system under study requires us to determine exchange-coupling parameters that within single-configuration density-functional pictures require the use of spin-unrestricted calculations. It is often noted that such techniques lead to spin contamination and a broken spin symmetry. However, the eigenstates resulting from Heisenberg Hamiltonians, constructed from broken symmetry methods, retain the symmetry of the system and restore the expected symmetry of the resulting spin eigenstates of the system. The other type of spatial symmetry relevant to the system here is the fact that the 207-atom Co_2_ moieties almost have a 3-fold symmetry axis which, if not locally broken by the *NC*_5_ *H*_5_ groups and counterions, could accommodate extremely high magnetic anisotropy. Throughout this paper, we have annotated many of our references to symmetry with either ″(PGS)″ or ″(SS)″ (spin-symmetry) to prevent confusion unless we explicitly refer to spatial or spin in other ways.

## 3 Magnetic Anisotropies

The structural and magnetic properties of the MMBB hexagon are summarized in Tables 1 and 2.

**Table 2:** Properties of the Hexagonal MMBB System^a^

(NC_5_H_5_[Co])_6_	μ^*A*^	μ^*B*^	μ_*tot*_	MAE (K)	Gap (eV)
FM	2.62	2.51	18	59.6	0.64
AF	2.63	–2.52	0	59.3	0.65

(NH_3_[Co])_6_	μ^*A*^	μ^*B*^	μ_*tot*_	MAE (K)	Gap (eV)
FM	2.63	2.53	18	54	0.54
AF	2.64	–2.53	0	55	0.54

a The MAE reported corresponds to the second-order treatment. The spin density integrated within a sphere of radius 2.22 Bohr yields local moments in the range of 2.51–2.63 which is consistent with *S* = 3/2 cobalt ions . Intermolecular Co–Co distances are 4.36 Bohr.

As discussed in many places, one needs to choose an energy window to be used in the perturbative or exact-diagonalization method that is used for determining the magnetic anisotropy energy. For Mn_12_–Acetate, this energy window was found to be quite small, and the same holds for the hexagon. In Figure 3 we show that the magnetic anisotropy is insensitive to much smaller energy windows than those we have employed in this work.

**Figure 3:**
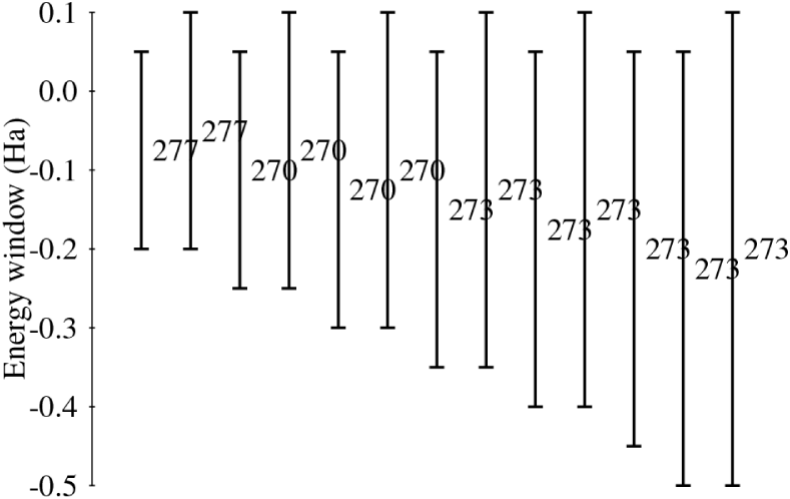
Energy windows used in the evaluation of the magnetic anisotropy energy (MAE) in K for the ferromagnetic trianionic case, which has small HOMO/LUMO gaps. Each vertical bar represents a single calculation. Horizontal ticks denote the lower and upper bounds of the energy window; energy values are read from the vertical axis. The corresponding MAE is shown at the center of each bar.

The 864-atom toroid was optimized within the symmetry constraint of the S_6_ symmetry. Using the methods outlined for calculations of molecular magnetic anisotropy energies,^15^ we find that, to the second order, the magnetic anisotropy Hamiltonian is given by $H=DS_{z}^{2}$ as is expected for a system with a 3-fold axis. Higher-order effects can be approximated by performing exact diagonalization, or noncollinear calculations^8^ rather than the perturbative method described in ref^15^.

We now turn to the question of what type of quantum behaviors this molecular toroid could provide. Spin-electric molecular magnets are traditionally composed of an equilateral triangle decorated by only one identical metal center (often spin 1/2). In a standard spin-electric, each of the identical centers carries a nonzero net moment that can preside in either a spin-up or spin-down configuration. If the two spins at the base of a triangle are antiparallel to the spin at the vertex of the triangle, the electrons on the base of the triangle are able to partially migrate to the vertex, which leads to the spontaneous appearance of an in-plane dipole, which is of course allowed by symmetry once the spins are antiparallel. Since the system described here is composed of interpenetrating triangles, the possibility of using one of the triangles as a spin-electric is worthy of consideration. The easiest way to map the tiling scheme proposed here onto a spin-electric would be to use MMBB that has an odd number of centers on each site. In such cases, regardless of on-molecule exchange coupling (Ferro- or Ferri-magnetic), one could directly construct spin-electric systems that are qualitatively similar to those proposed to date. Alternatively, for the AF MMBB, with an even number of metal centers studied here, a single-electron or single-site collective spin flip would yield a triangle with nonzero net spin and, as shown in Table 1, would change the energy of the triangle due to the change in energy of the interactions between the three on-site dipoles that are normal to the plane. In direct analogy to a standard spin-electric, some change in transverse electric dipole would result due to the changes in spatial orthogonality constraints and the breaking of the 3-fold symmetry axis. However, it is more likely that a strong spin-electric effect would arise due to charge transfer that attempts to counteract changes in interactions between the dipoles that are normal to the surface.

## 4 Summary and Conclusions

To summarize, Hooshmand et al.^10^ studied the fundamental building block of a Co_2_ molecular magnet that was synthesized by Maass et al.^11^ While the bulk of the Co_2_ moiety had a 3-fold symmetry axis, the fundamental building block presented no symmetry due to the pyridine termination of one of the Co ions. Their study included calculations on the intrinsically 3-fold system and showed possibilities for huge magnetic anisotropies that changed dramatically when the coupling of Co ions changed from AF to FM (due to an electronic level crossing). Here, we have studied the possibility of constructing this building block as a single isolated hexagonal ring and have shown that chiral symmetry will lead to a magnetic anisotropy Hamiltonian that is devoid of first-order transverse terms. However, the strong dependence of the anisotropy, electronic structure, and spin coupling identified in earlier studies on the single Co_2_ moieties remains. These couplings and the coexistence of permanent dipole moments raise the possibility of designing interesting spin- or magneto-electric quantum systems. We also note that for the aspirational case, for which the post-SCF anisotropy is 415 K, the anisotropy is three times larger, with the opposite sign, at second order, which means there may be very interesting higher-order effects and which argues further for devices based upon changes in the magnetic anisotropy controlled by field-induced level crossing. The isolated MMBB studied here does not quite have a 3-fold symmetry axis. This leads to a second-order single-spin magnetic anisotropy Hamiltonian that exhibits an E-term, for the isolated systems, that is not present in the crystal. We have demonstrated one way to deposit this MMBB on a two-dimensional hexagonal lattice that restores overall uniaxial symmetry and have explicitly shown, with density-functional calculations, that the second-order anisotropy Hamiltonian does not show an E value. A combination of permanent dipole moments associated with the MMBB and a spin-ordering-dependent energy of a mobile electron^10^ allows for enhanced exchange coupling that is similar to mechanisms observed in spin-electric systems.

While the pyridine-terminated system, which corresponds to the as-synthesized system, provides stable local moments on the two different cobalt ions and a large HOMO/LUMO gap, it does so by breaking a symmetry between 2-fold *Co*(3*d*-*e*_*g*_) electrons which limits the possibility of obtaining the huge anisotropies in a carefully crafted *NH*_3_-terminated mimic of the experiment. While this near-degeneracy is indeed maintained in the aspirational case *A-*2, it seems that the best way to alleviate the additional complications that occur due to accidental degeneracies between different Co_2_ electrons is needed for further predictive progress. The FLOSIC method^51,52^ has demonstrated some success in more accurately determining the local electronic structure of transition-metal ions^53^ but is likely very important to include the complex generalization, introduced by Withanage et al.,^54^ to address this type of system and avoid the loss of orbital symmetry degeneracies.

The point here is that the easiest way to achieve high magnetic anisotropy in transition metal systems is to have a half-occupied 2-fold state as the HOMO level. In such cases, the occupied d-electron is in a *Y*_*lm*_ state with *m* = ±2 or *m* = ±1, and it carries a permanent orbital moment that couples to spin at the first order, rather than the second order.^55^ In this system, the counterion and the pyridine ion break the local point-group symmetry and cause local Jahn–Teller distortions which quench the very strong anisotropy. cFLOSIC, as developed by Withanage et al.,^54^ allows for the emergence of open-shell half-occupied states that can be potentially enhanced due to the additional possibility of non-JT solutions with permanent orbital moments.

To test the effect of basis sets, we have varied the number of contracted orbitals on the atoms and found that the MAE is insensitive to the basis set. We used case A-2 for these basis set tests. When the Co-basis is changed from (5 s-type, 3 p-type, and 3 d-type) to (7 s-type, 5 p-type, and 4 d-type), the magnetic anisotropy does not change. Similarly when the choice of long-range to moderately long-range single Gaussians on the oxygen and nitrogen is varied, there are no appreciable changes in the anisotropy. For the interesting anion result, where the magnetic anisotropy was found to be 273 K, the result is independent of the number of occupied and unoccupied states used in the perturbative expression.

The confirmation of such a tiling structure may be experimentally validated by noting the *absence* of Berry-phase oscillations that have been predicted by Garg^56,57^ for cases where transverse anisotropy exists. The magnetic behavior of this twisted surface structure is expected to allow for both quantum tunneling of magnetism and spin-electric behaviors. As a final remark, we point out that much has been standardized in public and commercial quantum chemistry codes, and it is now common for researchers to use standard numerical methods and basis sets and compare functionals to determine best practices for quantum calculations that are fully consistent. In this paper, we have reached back to a method, based on the use of point-group symmetry, to build uniaxial hexagonal structures from asymmetric building blocks. The efficacy of this method is exemplified by the early work of Karl Johnson and coworkers in applications to building fullerene tori.^1^ With recent interest in chiral systems for chemical applications, we hope that this work encourages others to employ such strategies to more efficiently perform calculations on a large variety of chiral systems.

## Supplementary Material







## Notes

information on how read-in NRLMOL basis and convert to other formats if desired. (TXT)
the coordinates, in Jmol-readable format, of the NH3 terminated structure. (XYZ)
the coordinates, in Jmol-readable format, of the Pyridine terminated structure. (XYZ)
